# Canadian Paramedic User Requirements for Speech Recognition-Aided Documentation

**DOI:** 10.1055/a-2969-2253

**Published:** 2026-10-07

**Authors:** Desmond Hedderson, Karen L. Courtney, Helen Monkman, Ian E. Blanchard

**Affiliations:** 1 8205 School of Health Information Science, University of VictoriaVictoriaCanada; 2 Department of Community Health Sciences 2129 Cumming School of Medicine, University of CalgaryCalgaryAlbertaCanada; 3 3146 Emergency Medical Services, Alberta Health ServicesEdmontonCanada

**Keywords:** user requirements, paramedicine, clinical documentation and communications, electronic health records and systems, continuity of care, paramedicine informatics

## Abstract

**Objective** Developing technology may assist paramedics in the competing tasks of providing life-saving care and accurately documenting that care. The objective of this study was to identify and classify paramedic user requirements for speech recognition (SR)-aided documentation.

**Methods** This was a two-phase, mixed-methods study. Phase 1 involved a web-based questionnaire distributed to paramedics across Canada. Phase 2 consisted of opt-in one-on-one interviews to gain further insight into the survey results. Descriptive and associative statistics were used to identify which features respondents deemed most important, which were classified into five categories based on questionnaire results. Thematic content analysis of the qualitative data was used to understand the quantitative rankings.

**Results** A total of 323 respondents completed the questionnaire. Overall, 86% of respondents ( *n* = 277) had a positive perception of SR. Respondents rated the following 10 features as most important: Patient Interaction, Compliance, Support, Durability, Training, Accuracy, Continuous Improvement, Training Support, Dictation, and Editing. Themes from 14 interviews described the role of technology in the future of paramedicine, adoption enablers and barriers, and SR benefits. Joint displays were used to gain further insight into the quantitative ratings, including the possibility that the presence or absence of these features could affect adoption.

**Conclusion** These prioritized features highlight the unique practice context of paramedicine. While this was a Canadian study, the results may be applicable to other similar paramedicine practice systems. This work provides a robust list and classification of SR user requirements for Canadian paramedics.

## Background and Significance

Paramedics must accurately document patient assessments, past medical history, medications, scene assessments, interventions, and any care provided in an electronic patient care report (ePCR), often in an uncontrolled prehospital environment. 1 The ePCR is intended to relay all important patient data to the receiving facility and practitioners. 2 The demographic, geographical, social, and clinical data collected in the ePCR are essential to emergency department (ED) staff decision-making and to other interested parties. 1 2 3 The patient information captured in the ePCR is valuable for ensuring continuity of care and assists ED staff in making appropriate decisions regarding treatment and initial diagnosis. 4 However, because paramedics prioritize patient care, the workload and documentation burden can delay completion of the ePCR. Consequently, the ePCR is not always available during ED decision-making. 5 Moreover, when the ePCR is completed after arrival at the hospital or after another response, paramedics must recall details retrospectively, which can compromise the ePCR accuracy. 6 Unsurprisingly, patients are regularly delivered with missing or omitted information. Not only can ED physicians be frustrated by delays in obtaining ePCRs, 7 8 but these delays can also have consequences for patient care.

Approximately 25 to 40% of adverse events are attributable to information gaps (i.e., where crucial information is unavailable to providers during care) in the ePCR. 2 9 10 These information gaps can also lead to extended ED stays. 7 While verbal paramedic-to-ED staff handovers were implemented to capture critical patient information, numerous barriers impact their efficacy. 11 12 13 Despite efforts to improve these handovers using mnemonic devices, 11 14 almost all transfers (99.4%) were found to be deficient in either structure (i.e., the order of information) or scope (i.e., the extent of information). 11 14 15 Given that verbal handovers have only been minimally effective in reducing patient information gaps, 11 15 16 more reliable and effective methods for relaying important patient information must be pursued.

The use of speech recognition (SR)-aided documentation by physicians has been shown to reduce charting time, produce more complete notes, and be less error-prone than typed charting. 17 Newer technologies, such as ambient artificial intelligence (AI) documentation that utilizes SR, have also been reducing documentation burnout. 18 SR-aided documentation in paramedicine may mitigate missing or omitted patient information resulting from paramedicine’s documentation challenges. SR-aided documentation has been well-studied in hospitals for decades; 19 20 however, limited work has been dedicated to SR in the prehospital context of paramedicine. While the additional noise in the prehospital milieu presents challenges, recent advancements in AI, natural language processing, and other technologies have made SR-aided documentation in paramedicine workflows more feasible. 21 22 23

Given the high costs and failure rates (between 40 and 70%) of health care technology implementation projects 24 in general, and the current lag in adopting digital documentation strategies in paramedicine, 1 for SR-aided documentation to be successful, it is critical to take concrete steps to improve adoption rates. Applying user-centered design principles can enhance the adoption of new technologies. 25 Understanding user requirements is an essential component of user-centered design and the first step in an iterative process to ensure alignment between users’ needs, the work they do, and the tools they use to accomplish their tasks. 25

## Objective

The objective of this study is to identify the user requirements of Canadian paramedics for SR-aided documentation.

## Methods

This research was designed as a two-phase, sequential, explanatory, mixed-methods study, comprising a questionnaire phase and an interview phase. 26 27 28 This approach had previously been used to explore the context of technology use and usability, providing further insight into the user requirements identified in the questionnaire. 27 28 This study was approved by the University of Victoria Research Ethics Board (identifier: 23-0450). To begin, a scoping review of the literature was conducted to identify preliminary user requirement categories. 29 These requirements included workflow integration, SR dictation, comfort, interoperability, minimal interface, word accuracy, decision support, battery life, noise reduction, and hands-free documentation. 29 Based on these requirements, market research, and existing technologies, a list of 45 potential features was developed.

### Recruitment

The questionnaire was distributed using regulatory bodies, professional associations, and alumni associations to reach the broadest cross-section of paramedicine practitioners across Canada. This included the Alberta College of Paramedics, British Columbia Emergency Health Services, the Paramedic Association of Canada, the Paramedic Association of Ontario, the Emergency Health Services of Nova Scotia, and other similar organizations. The organizations were provided with an information letter, an email template, and a survey link, which they in turn distributed to their respective members. The recruitment included actively registered Canadian paramedics, paramedicine students, former or retired paramedics, and leadership within paramedicine organizations.

### Questionnaire Phase

The initial phase of the study consisted of a web-based questionnaire that collected information on 45 prospective features of SR-aided documentation (e.g., medication timestamping, accuracy, dictation, patient interaction). The potential features included both technical and context/implementation requirements based on Sittig and Singh’s new sociotechnical model dimensions. 30 These dimensions included Hardware and Software, Clinical Content, Human–Computer Interface, People, Workflow and Communication, and Internal Organizational Policies, External Rules, and System Measurement and Monitoring. 30 Participants were asked to score the features on a scale of 1 to 5, from “least to most” valuable or important. Additionally, the questionnaire included open-ended questions to allow respondents to describe additional functionality and explain their potential feature rankings. Respondents were also given the option to define the five features they considered essential for improving adoption. A table of the original survey question, its short form and associated abbreviations is available in the **Appendix A** (available in the online version only).

### Interview Phase

The second phase of the study included a semistructured interview (30–60 min) conducted over Zoom by one author (D.H.), which was then transcribed using a denaturalized approach. 31 The interview guide was structured around the four-stage process described by Stanfield, consisting of objective, reflective, interpretive, and decisional questions. 32 The iterative guide approach allowed for follow-up clarifications and the inclusion of unforeseen questions that arose during the interview. Interview participants were selected from the pool of survey respondents who opted in for the interview to capture a range of diversity in gender, paramedicine role, geographic location, practice area, work experience, and specific and overall perceptions of SR-aided documentation. Data saturation determined the final number of interviews. A full selection matrix for interview participants is available in the **Appendix B** (available in the online version only). Transcripts were anonymized by removing names and references to the working environment and locations. One author (D.H.) used NVivo 14 to compile and code transcripts from interviews and open-ended question responses.

### Analysis

The questionnaire data were analyzed using descriptive and bivariate statistics to identify the most valued features. Only completed responses were used in the analysis, with incomplete or partial responses removed. The Likert ratings from each of the sociotechnical models’ eight dimensions were isolated, 30 and all quantitative analyses were completed in RStudio (version 2024.12.0+467, Posit Software). The qualitative data were analyzed using thematic content analysis. 33 This enabled analysis of interview transcripts and open-ended questionnaire responses to better understand feature rankings. Themes were developed based on the eight dimensions of the sociotechnical models, as well as recurring themes that emerged around user preferences and each of the 45 proposed features. 30 Results were then combined using joint display analysis, where possible. 34 The qualitative data were used to provide further context for the quantitative results.

## Results

The total number of questionnaire respondents was 534, with a 60% completion rate, yielding 323 usable responses for analysis. We received responses from all provinces in Canada, but not from any of the territories (i.e., Yukon, Northwest Territories, and Nunavut). The demographics of respondents aligned with those reported for paramedics in Canada in terms of age, gender, and area of practice 35 (see Table 1 ). There were 14 interviewees who represented a broad range of practice areas and locations, as well as years of paramedicine experience and overall perceptions of SR technology.

**Table 1:** Respondent demographics.

Respondent demographics	Count (percentage)
Province	
Newfoundland Labrador	11 (3)
Nova Scotia	29 (8)
Prince Edward Island	8 (2)
New Brunswick	48 (15)
Quebec	11 (3)
Ontario	59 (18)
Manitoba	13 (4)
Saskatchewan	32 (10)
Alberta	62 (19)
British Columbia	50 (15)
Gender	
Man	189 (59)
Woman	121 (37)
Non-binary	5 (2)
Prefer not to say	7 (2)
Self-described	1 (0.3)
Area of practice	
Urban	186 (58)
Rural	123 (38)
Remote	14 (4)
Age group (y)	
0–20	6 (2)
21–30	88 (27)
31–40	94 (29)
41–50	69 (21)
51–60	48 (15)
61+	18 (6)
Experience (y)	
0–5	95 (29)
6–10	61 (19)
11–15	38 (12)
16–20	41 (13)
21–25	29 (9)
26+	59 (18)
Overall perception of SR technology	
Positive	277 (86)
Negative	46 (14)

Abbreviation: SR, speech recognition.

All 45 features presented to respondents were combined and ranked based on their overall high importance or value rating (4 or 5), as shown in Fig. 1 . The features were ordered from most important or valuable to least important or valuable. Definitions of each feature that were presented to participants are available in the **Appendix A** (available in the online version only).

**Fig. 1:**
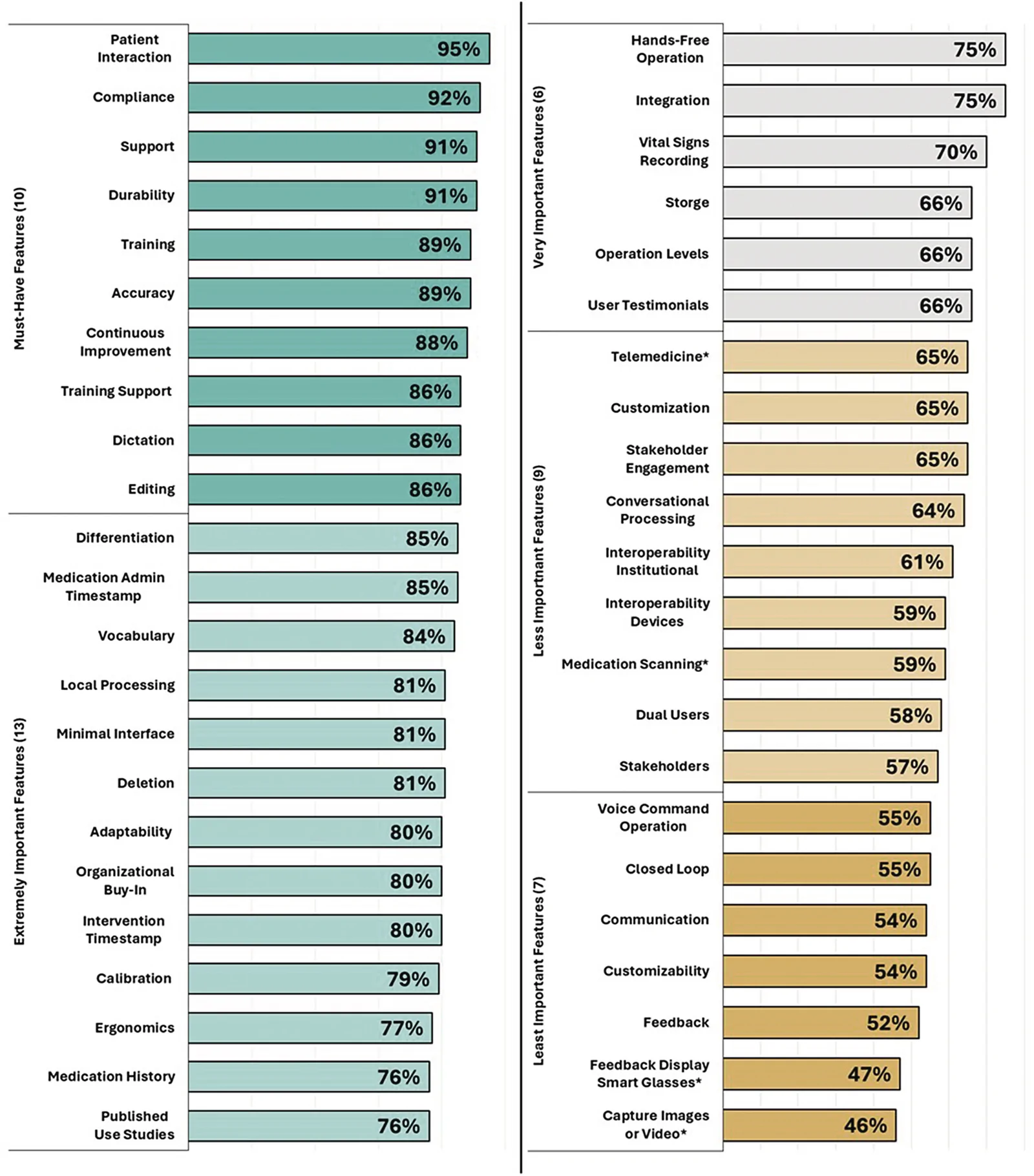
Features are classified into tiers by desirability. All provided features, those with the symbol “*” were listed as part of a potential smart glasses implementation. The graphic is divided at the median, with all values below it on the right.

The difference between the highest-value feature (95%) and the lowest-value feature (46%) was divided into five equal groups to classify feature desirability into tiers. Classifications included must-have features (86–95%), very important features (76–85%), important features (66–75%), less important features (56–65%), and least necessary features (46–55%).

The highest-rated features included Patient Interaction (95%), Compliance (92%), Support (91%), Durability (91%), Training (89%), Accuracy (89%), Continuous Improvement (88%), Training Support (86%), Dictation (86%), and Editing (86%). The definitions for each feature are available in Table 1 (available in the online version only). The highest-rated features also featured prominently in the paramedic interviews and in the open-ended answers from questionnaire respondents. Some participants and respondents identified that the feature would need to be present for them to consider adopting the technology. Other participants discussed how the absence or poor implementation of these features would deter them from adopting the technology. Some representative quotes from the study on the top five features are available in Table 2 .

**Table 2:** Joint display describing the highest-rated features from the interviews and free-text sections of the questionnaire.

Ratings	Quotes	Meta-inferences
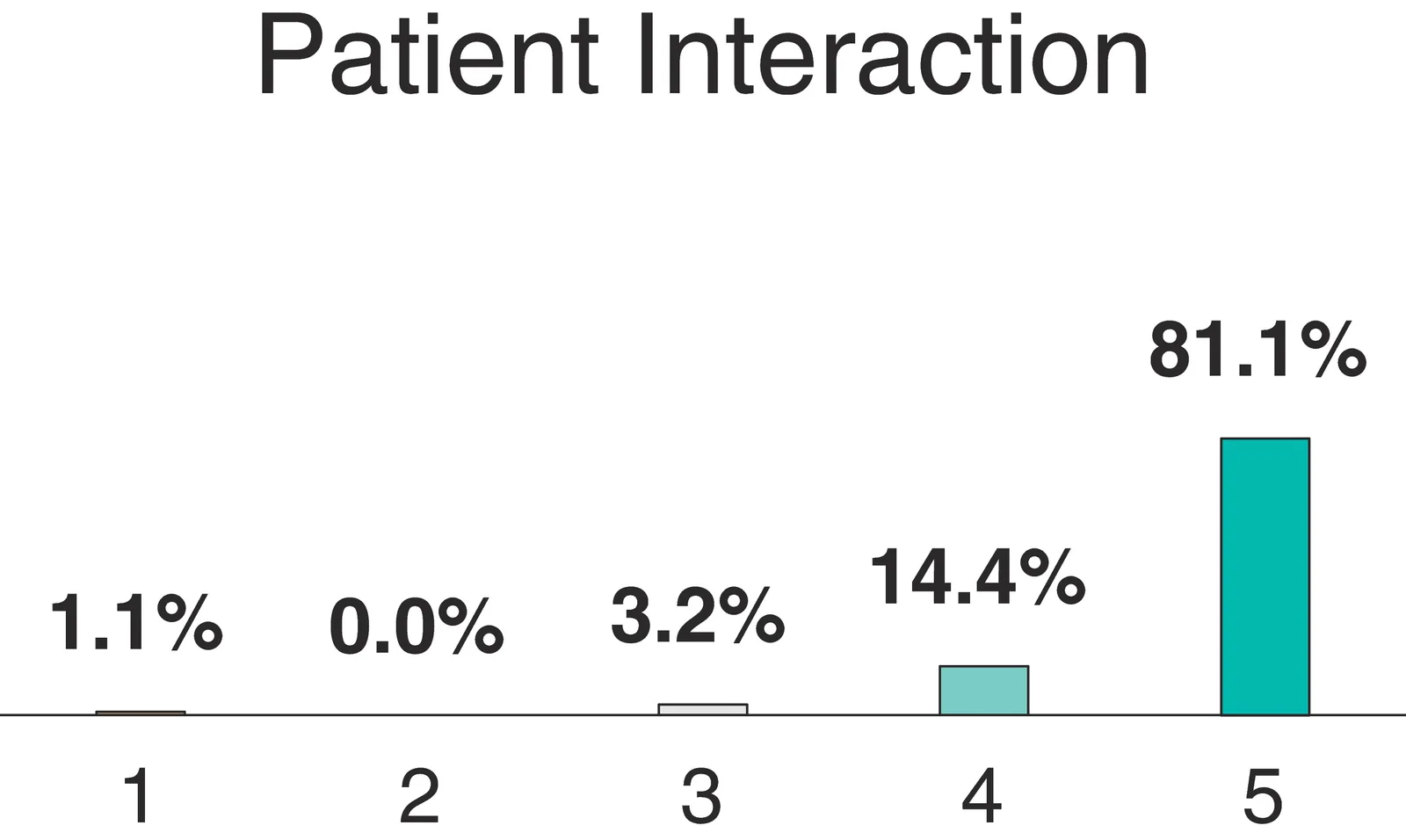	“Well, for me, it’s not interfering with patient interaction. Both the use of it (and the device itself).” – I #7 “If speech-recognition technology distracts from or otherwise compromises patient care, I would be less likely to use it.” – R #398	Paramedics value the importance of the patient interaction and care. Any technology introduced must be careful not to be intrusive to the patient relationship.
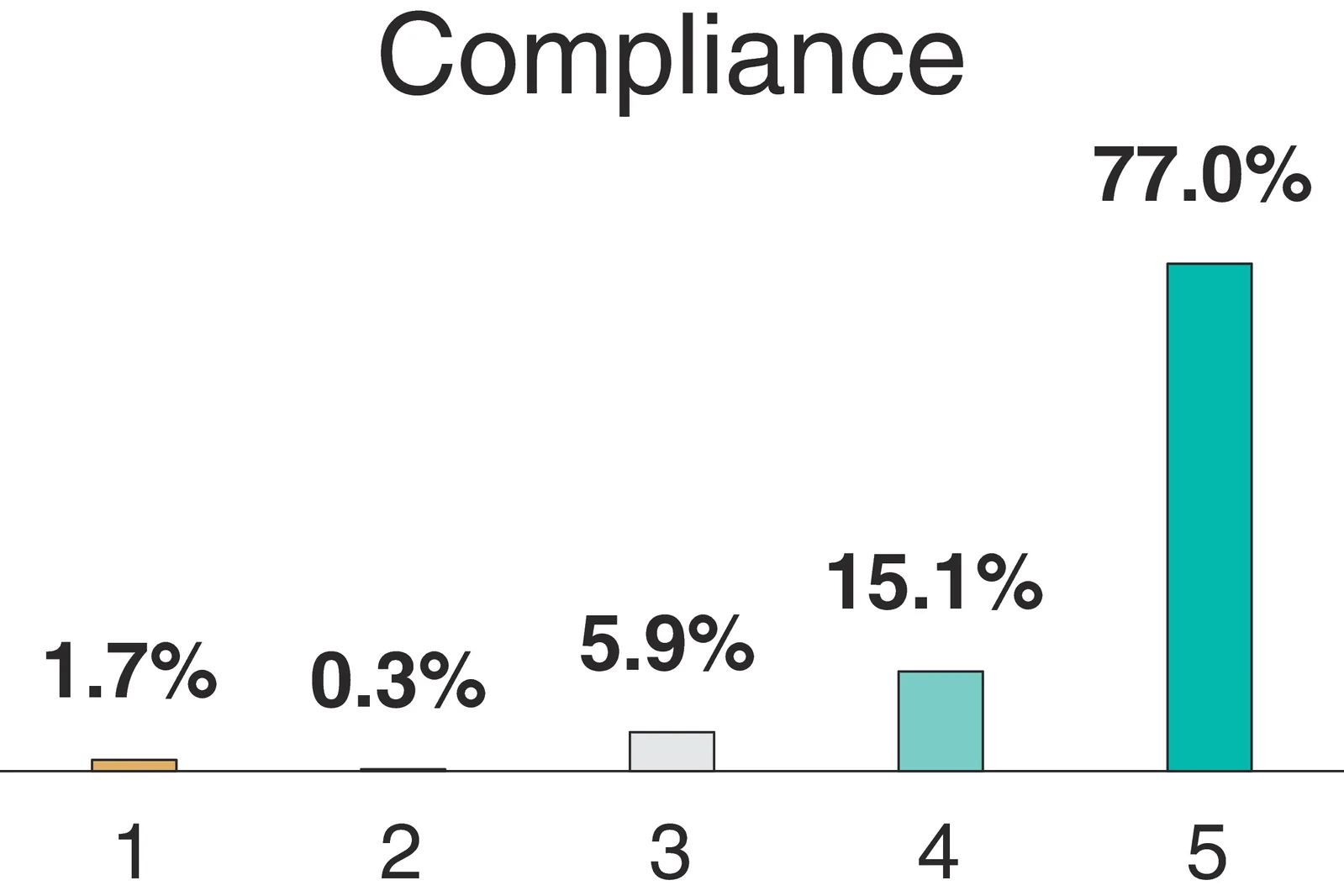	“I think the thing that would present the biggest barrier is getting city privacy legal and service, privacy and legal onboard with the like protective measures within the software and the device to prevent privacy breaches. Because I think that would be the first concern they would raise, and until it’s addressed to their satisfaction, there’s no chance of moving forward with it as a deployed device.” – I #4 “Privacy legislation is possibly the largest barrier and would need to be addressed specifically. When documenting (for example), other people could overhear what is being said into the device when they were not involved in the circle of care.” – R #363	Ensuring that patient privacy is protected is very important to paramedics. Ensuring that any device or software introduced meets all necessary legal requirements will be critical.
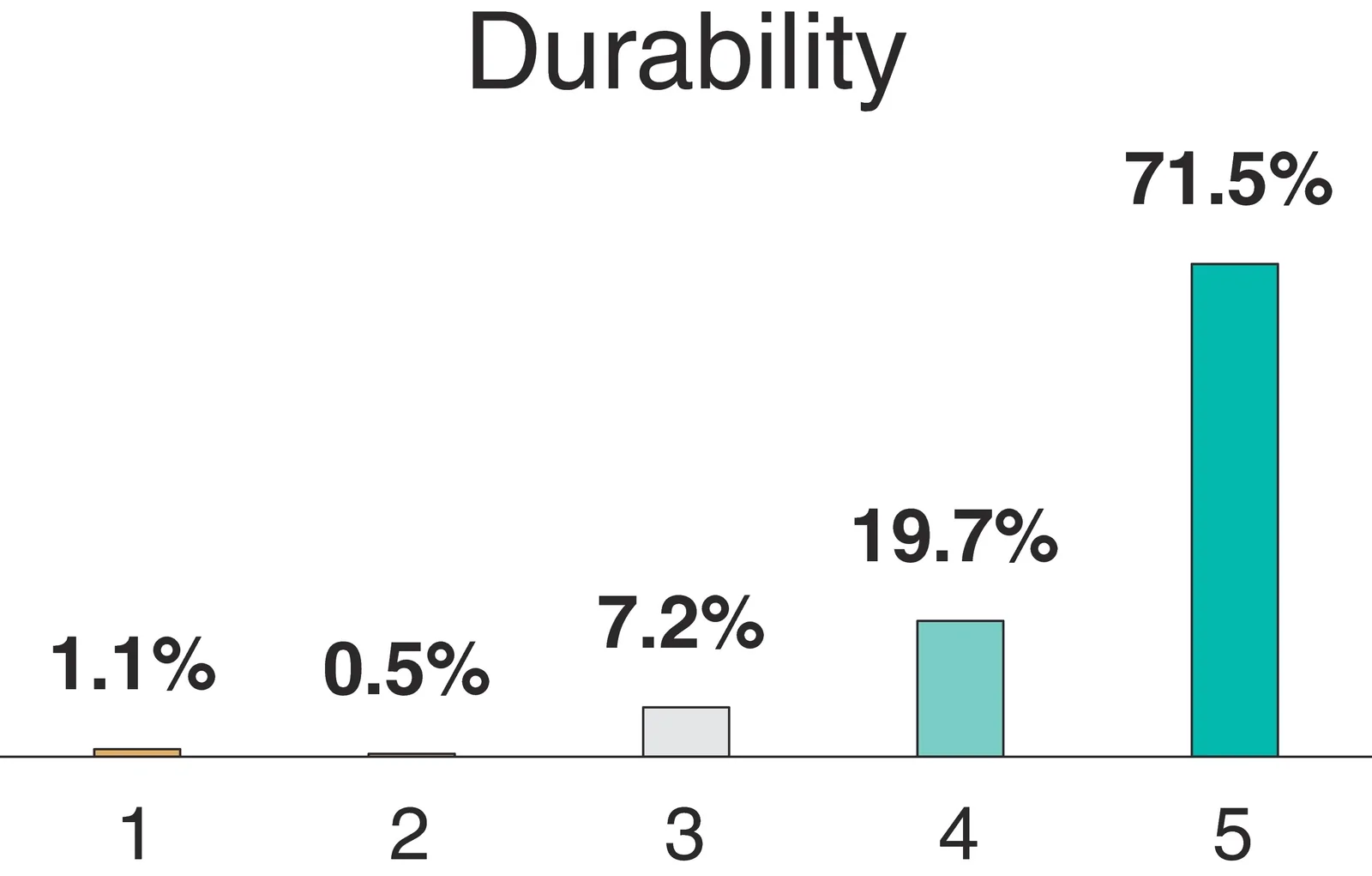	“Durability, paramedics are tough on equipment. Oh, my stars. We’ve had stuff run over like thousands of dollars, so I could just see this. Like we have to hand off a piece of equipment, so if I’m using these safety devices, this radio, this backup safety device, this cell phone, I have to make sure that I’m leaving it for the next crew that’s coming in and even doing that like I think even just the durability (for constant multiple users), we would definitely (need it to be Durable).” – I #10 “I am concerned that the technology will have a hard time functioning consistently in our work environment.” – R #17	Paramedicine workflows necessitate a robust hardware design. The need to design the system for multiple users, long battery life (12+ hour shifts), to function in harsh conditions and to be easily sanitized for the next users is a high priority.
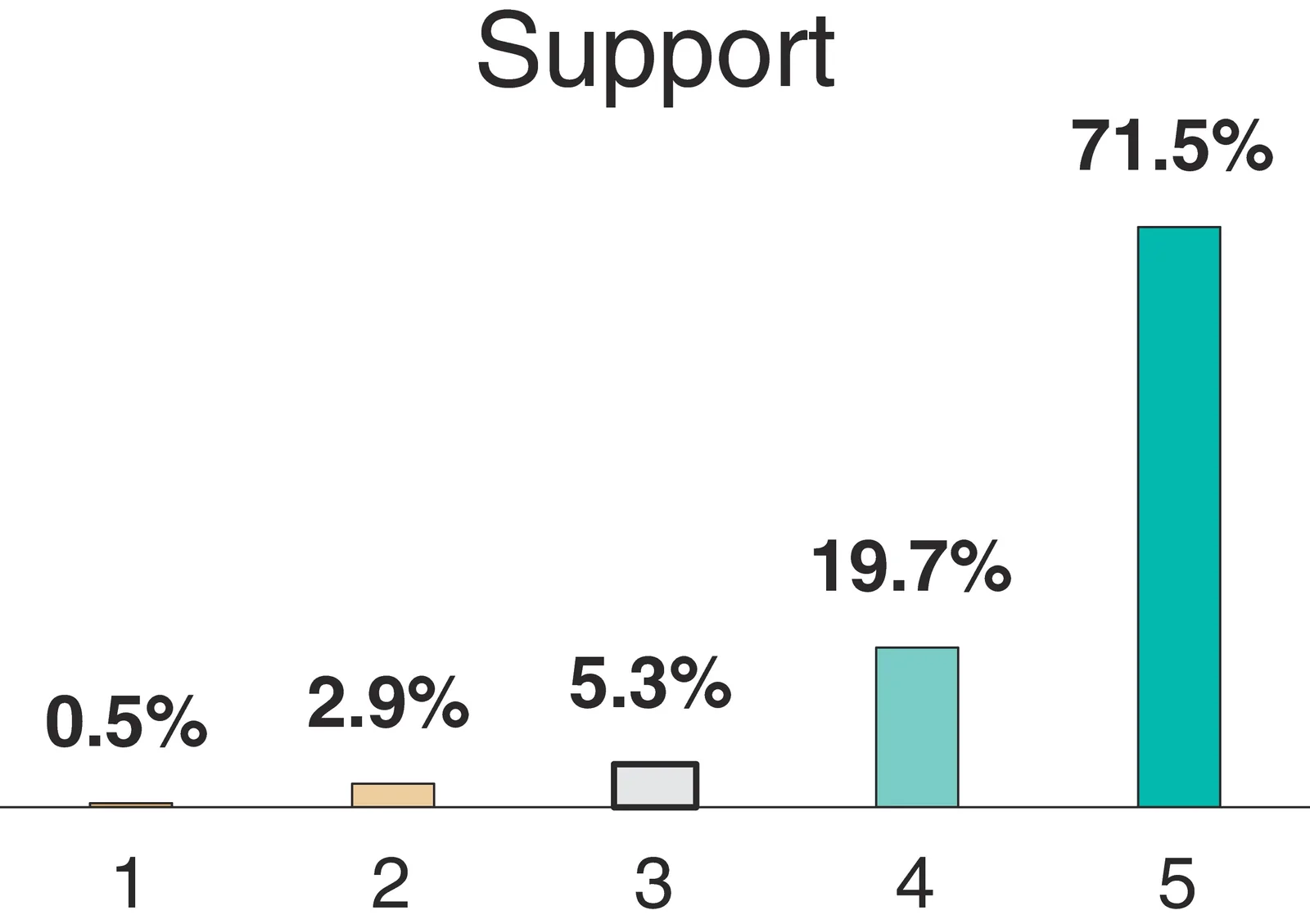	“With some of our paramedics who are less technologically adept, that seems to be a lot of my day-to-day, even just getting people’s email passwords reset or accessing their email app or accessing their (online) schedule, all of that seems to be very challenging. So we would need a robust support system where either a supervisor could ask on behalf of a paramedic or the paramedic could receive that support very quickly and seamlessly. Because a lot of our paramedics, despite kind of how our society is moving, surprisingly, they still just think they only prioritize learning about technological advances that they want to learn about, and so when it’s associated with work, they don’t necessarily make that connection or put in that time or that effort.” – I #10	Access to quick support for troubleshooting is critical for paramedics. Given the time-sensitive nature of paramedicine, spending time trying to reach technical support is problematic.
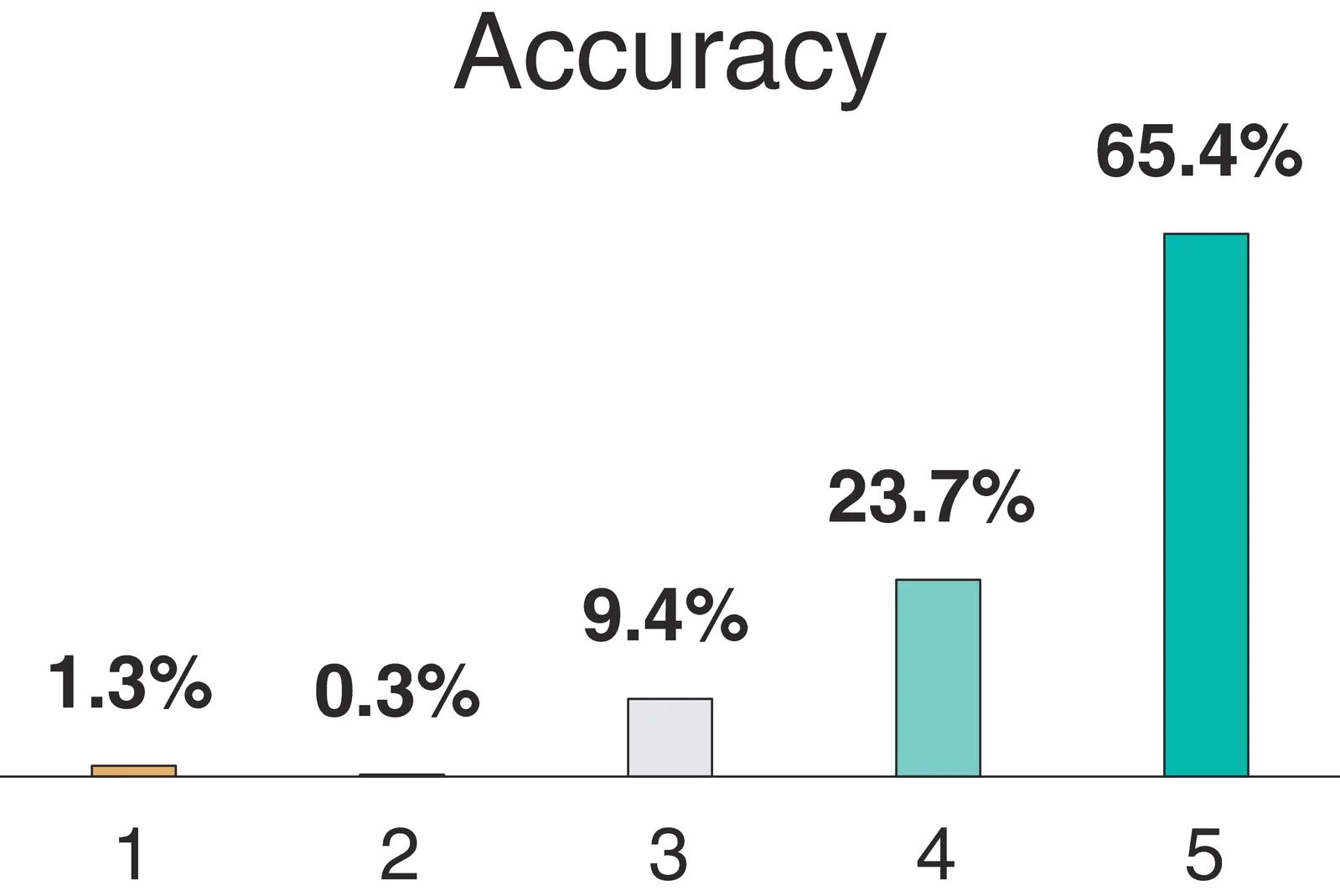	“So I think for me, the biggest thing would be, yeah, comprehension and clarity, but then also if I think that if it was transcribing my speech and it was incorrect would be very frustrating and very discouraging. I wouldn’t want to use the technology. I would find it just easier to just write it out myself.” – I #6 “The system will save the most time when it makes the least mistakes possible; otherwise, I’ll just have to do a ton of editing afterwards.” – R #161	Paramedic vocabulary is unique. There are many acronyms, short forms and abbreviations in use, similar to, yet unique from the rest of health care. Ensuring that the SR documentation software has high word accuracy and accounts for paramedicine-specific vocabulary is important to users.

Abbreviations: I, interviewee; R, respondent; SR, speech recognition.

Note: The chart of ratings shows the percentage of total selections on a scale of 1 to 5 (1 = least valuable to 5 = most valuable; *n* = 323).

The qualitative findings were analyzed to support and provide context for the quantitative findings, thereby fully exploring the user requirements. It was identified in both the open-ended questions and during the interviews that either the presence or absence of certain features would be critical to adoption. Each of the top-rated features was mentioned in either the open-ended questions, interviews, or both. The supporting evidence is included in **Supplementary Table 1** (available in the online version only) *.* Some novel features, not included in the list of 45, did appear in the analyses of the open-ended question and the interviews. These included live language translation (seven mentions), clinical decision support (five mentions), hands-free interaction with mapping systems (two mentions), AI or large language model “virtual doctor” assistance (two mentions), ability for smart glasses to do thermal scans (one mention), and the ability to directly upload patient data to the hospital electronic health record (one mention).

Themes of inevitable technological change, innovation in paramedicine, and the use of technology to improve paramedicine emerged throughout the interview process and underscored the idea that paramedics recognize that technology will play a larger role in the future. The qualitative data also identified the benefits that paramedics perceived SR-aided documentation would have, categorized as input, output, and system benefits. These ranged from improving accessibility to improved patient care. The barriers and enablers affecting the adoption of the SR-aided documentation system are categorized as external, internal, and practice factors. The enablers included paramedicine-focused design and adequate training, while the barriers included privacy concerns and employer distrust.

A total of 167 respondents answered the optional question to identify the five features they believed were essential for adoption. Respondents listed a total of 40 different features at least once. The top five most suggested as a minimum set were Dictation, Accuracy, Durability, Ergonomics, and Interventions Timestamping. Interventions Timestamping was defined as using voice commands using SR to record details about the intervention. The count for each of the minimum set features is displayed in Fig. 2 .

**Fig. 2:**
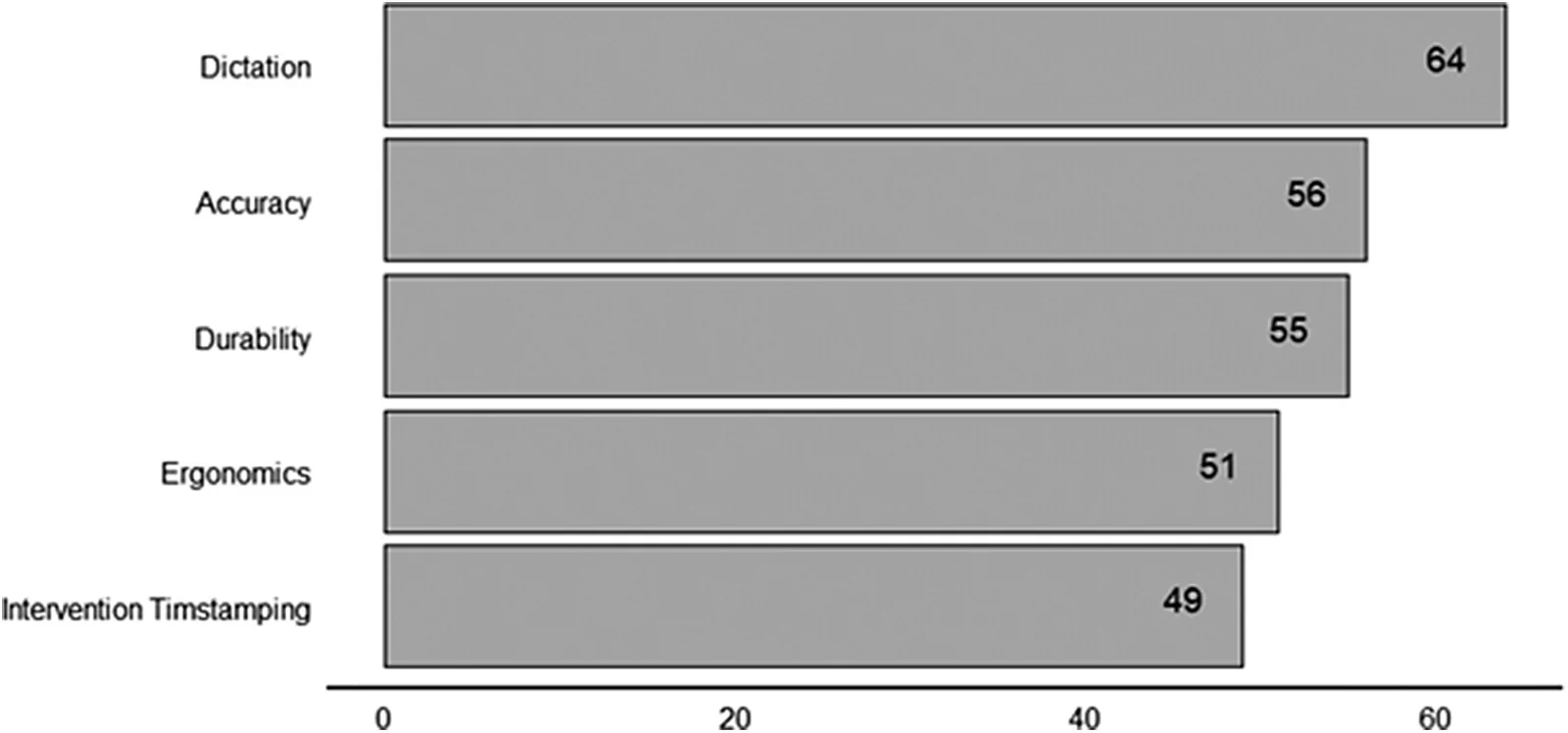
The top five mentioned features were the minimum requirements for adopting a speech recognition documentation system ( *n* = 167). Value is the number of times that the feature was selected as a minimum requirement.

## Discussion

This research aimed to identify user requirements and priorities of Canadian paramedics for SR-aided documentation. Respondents have indicated that any SR-aided documentation must not interfere with patient interactions, be medically and legally compliant, be durable, have available technical support, and have a high word-accuracy rate. The features were then classified into tiers based on individual rankings to identify which features paramedics valued most. The tiers included must-have, extremely important, very important, less important, and least important features.

The highest-rated (extremely—very important or valuable) features reflect the nature of paramedic work in Canada. Working in unpredictable, hectic conditions that span urban environments, rural areas, and remote communities underscores the need for durable, comfortable devices with robust local processing capabilities. The requirements also reflect paramedics’ professional commitment to patient care, safety, and comfort. This influences the desire for an accurate device and technology to ease documentation burden, without interfering with the paramedic–patient relationship or compromising compliance with all medicolegal requirements. Other health care professionals, physicians, and nurses have identified similar requirements. For example, training (the quality and quantity of training provided) and organizational support for training (time allotted, paid vs. unpaid learning) were identified as requirements for SR-aided documentation. 36 37 Nurses also identified the need for a highly accurate system that allows control of the output and final edits. 37 While most requirements were similar, paramedics appear to place greater value on different aspects of the technology, such as device durability and patient interaction, and lower value on interoperability and integration.

Notably, the features that were smart glasses-based all ranked in the lowest categories (less important—unnecessary features), which could be a consequence of the novel nature of smart glasses and the lack of familiarity with the technology, 38 or that paramedics place more emphasis on things they know work rather than untested technology. The features listed included both technical requirements and contextual or implementation requirements. In alignment with Sittig and Singh’s new sociotechnical model, the features listed accounted not only for technical system aspects but also for organizational policy, regulation, and system monitoring. 30 The top “must-have” features included selections from both technical categories (Durability, Dictation, Accuracy, and Editing) and context/implementation categories (Patient Interaction, Support, Training, Training Support, Compliance, and Continuous Improvement). It is worth noting that several implementation categories ranked higher than the technical categories. This highlights that implementation and organizational support for SR-aided documentation are crucial considerations for adoption. 30 This suggests that implementation must be carefully planned and not an afterthought. More work is needed in this area to develop optimal implementation strategies that can be used across organizations for guidance.

While this work was done in Canada, similarities with paramedicine practice models in other countries, such as the United States, the United Kingdom, and Australia, may make these findings generalizable beyond the Canadian context. These systems operate similarly and face similar challenges. While the United Kingdom and Australia operate professionally autonomous systems within socialized medicine, and the United States operates generally physician-directed, privatized systems, 39 they all operate in resource-constrained, uncontrolled environments, which will have similar documentation and technology needs. Paramedicine is a young profession with unique challenges, needs, and working conditions. The need for specific solutions for paramedicine is clear. However, little research has been conducted to understand paramedics’ technology needs, and this study represents a small step in the burgeoning field of paramedicine informatics.

### Future Research

More paramedicine-focused and led research will ensure that informatics tools, data standards, and workflows are grounded in the real-world clinical, operational, and contextual needs of paramedics rather than being adapted from other health care settings. 40 It is understood that having an in-depth understanding of requirements can help produce technologies that are more readily adopted by users. 41 42 Currently, there is a paucity of user requirement studies on SR-aided documentation in paramedicine, and further large-scale studies are needed. 29 For example, there is a need for research demonstrating that SR documentation meets the accuracy standards required by paramedics. One persistent perception is that SR documentation is not sufficiently accurate for medical documentation. Designing a study that could definitively demonstrate whether the accuracy of SR documentation meets or exceeds paramedics’ needs would be beneficial for informing those perceptions. Developing a paramedic-specific lexicon for SR documentation would also be a valuable area of research. 36 Participants identified that an SR documentation system would need to recognize the terms and shorthand used exclusively in paramedicine. Exploring the novel features mentioned by participants would also be valuable, especially the live language translation and clinical decision support possibilities.

### Limitations of the Study

The survey aimed to gather a substantial amount of information. This led to the survey being lengthy and time-consuming to complete (estimated at 20 min), which may have contributed to a significant attrition between those who began the questionnaire but did not complete it. The survey was also designed so that the demographic questions were at the end of the survey. This design choice resulted in many incomplete survey responses being unusable for further analysis involving demographics.

A second limitation was the distribution and response to the survey. Professional associations, employers, registrars, and other paramedic-specific groups were used as pass-throughs to distribute the survey. This meant the survey distribution relied heavily on the membership’s engagement with the organization and the organization’s willingness to promote the survey. The survey was also voluntary, with no compensation offered to respondents. This may have led to a nonresponse bias, 43 as most respondents had a keen interest in the subject, suggesting we missed the portion of the population that views it negatively or holds no strong opinions.

## Conclusion

The results of this study suggest the majority of respondents have a positive perception of SR technology, but some features are more important than others. The top “must-have” features included selections from both technical categories (Durability, Dictation, Accuracy and Editing) and context/implementation categories (Patient Interaction, Support, Training, Training Support, Compliance, Continuous Improvement). Qualitative data underscore that the presence or absence of certain features would be critical to adoption. This categorized list can be used by developers to tailor products and focus on high-value features, by paramedic administrators to ensure that support and policies are in place, and by researchers to design and execute further research on SR-aided documentation in paramedicine.

**Clinical Relevance Statement**

This study identifies the features paramedics consider essential for SR-aided clinical documentation. These findings can inform the design and implementation of electronic documentation systems that better align with paramedic workflows and patient care continuity. Electronic documentation systems incorporating these requirements may improve adoption, efficiency, and clinician–patient interaction in paramedicine.

**Multiple-Choice Questions**

Question 1: What is the primary purpose of identifying user requirements before implementing SR systems in paramedicine?

- To ensure the technology complies with hardware procurement standards
- To align system design with the workflows and needs of paramedics
- To eliminate the need for electronic patient care records
- To standardize all clinical decision-making processes
  **Correct Answer** : The correct answer is option b. Considering what the frontline clinical staff need to adopt the system into their clinical workflow is critical.

Question 2: Which challenge of paramedic documentation is SR technology most likely intended to address?

- Limited paramedic scope of practice
- The need to balance patient care activities with documentation requirements
- The lack of electronic documentation systems in health care
- The absence of standardized clinical terminology
  **Correct Answer** : The correct answer is option b. Paramedics face competing pressures when providing patient care.

## Supplementary Material

Ergänzendes Material Supplementary Material
