# Supplementary material for: Canadian Paramedic User Requirements for Speech Recognition-Aided Documentation
Source: Appl Clin Inform. 2026 Oct 7;17(4):873–81. doi: 10.1055/a-2969-2253 (PMC13645478; doi:10.1055/a-2969-2253)
Supplement: Supplementary file 1 [file 10-1055-a-2969-2253_29759037.pdf]

Thieme

Supplementary Table S1 Joint Display describing the highest rated features from the interviews and free text sections of the questionnaire. The chart of ratings shows the percentage of total selections on the 1-5 (1-least valuable to 5-most valuable) scale (n=323). Note: I = Interviewee; R =Respondent.

**Feature (%)** ***Description***	**Ratings**	**Quotes**	**M**
**Patient Interaction** (95%) *The device used to interact with speech recognition must not interfere with patient interaction.*	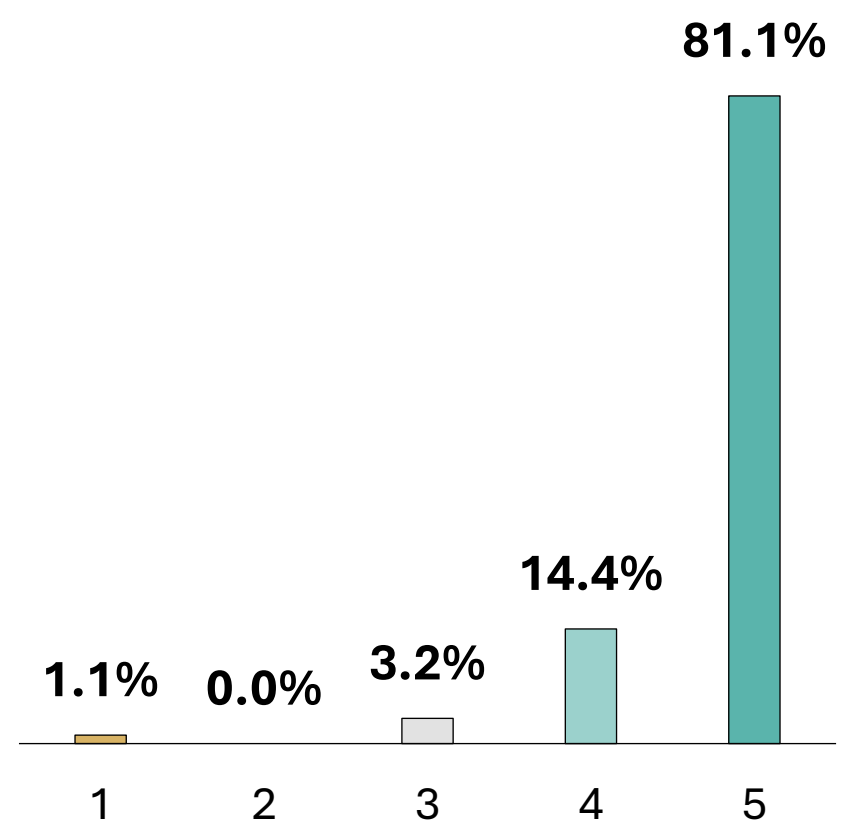	“Well, for me, it's not interfering with patient interaction. Both the use of it (and the device itself.)” – I #7<br><br>“If speech-recognition technology distracts from or otherwise compromises patient care, I would be less likely to use it.” - R #398	Par ir pati cai int (intr
**Compliance** (92%) *The speech recognition device meets all required medicolegal requirements for recording audio and video of patients.*	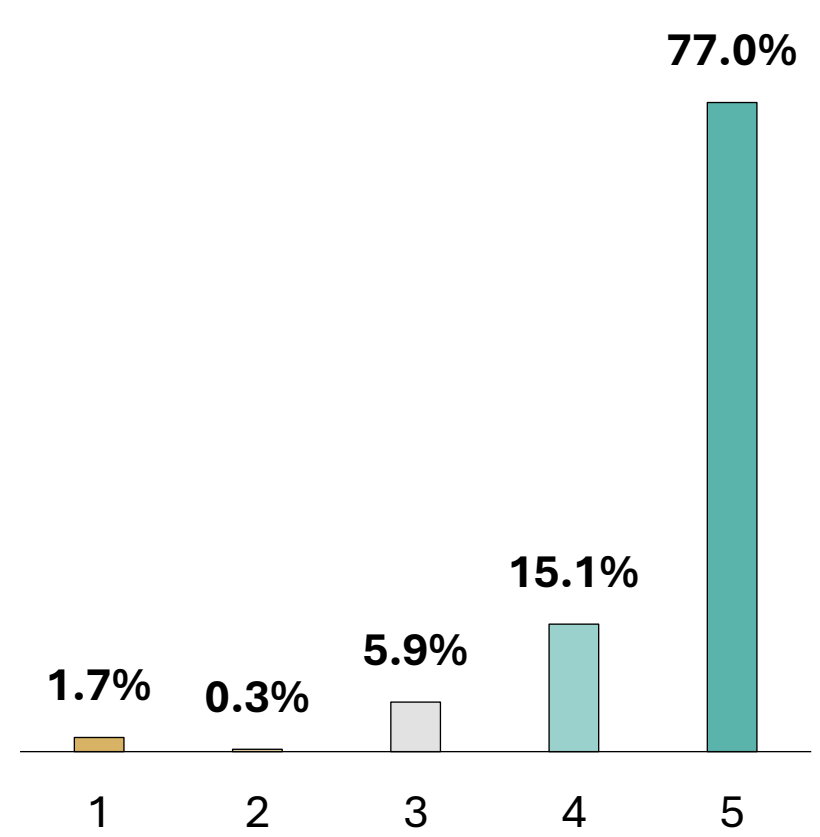	“I think the thing that would present the biggest barrier is getting city privacy legal and service, privacy and legal onboard with the like protective measures within the software and the device to prevent privacy breaches. Because I think that that would be the first concern they would raise, and until it's addressed to their satisfaction, there's no chance of moving forward with it as a deployed device.” – I #4<br><br>“Privacy legislation is possibly the largest barrier and would need to be addressed specifically. When documenting (for example), other people could overhear what is being said into the device when they were not involved in the circle of care.” – R #363	En: priv v par tl so me lega

**Durability** (91%)<br>*Sturdiness of the device, ability to easily sanitize, battery life, weatherproofing, etc.*	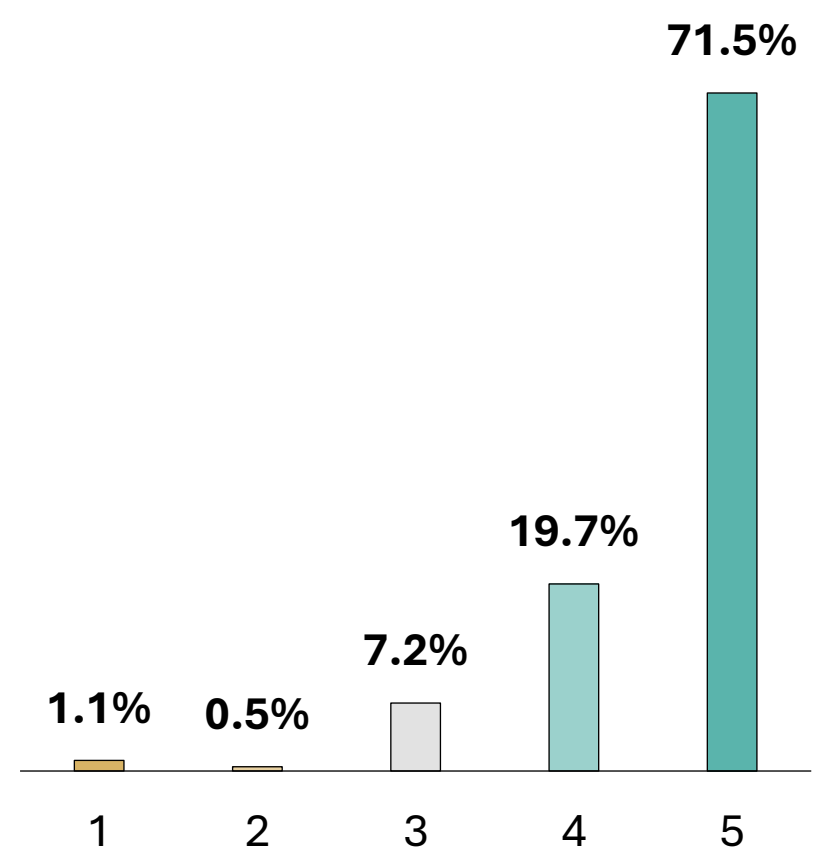 	"Durability, paramedics are tough on equipment. Oh, my stars. We've had stuff run over like thousands of dollars, so I could just see this. Like we have to hand off a piece of equipment, so if I'm using these safety devices, this radio, this backup safety device, this cell phone, I have to make sure that I'm leaving it for the next crew that's coming in and even doing that like I think even just the durability (for constant multiple users), we would definitely (need it to be Durable)." – I #10<br><br>"I am concerned that the technology will have a hard time functioning consistently in our work environment." – R #17	Para<br>ne<br>har<br>n<br>sy<br>use<br>(1<br>f<br>co<br>eas<br>ne
**Support** (91%)<br>*Easily accessible technical support for troubleshooting issues.*	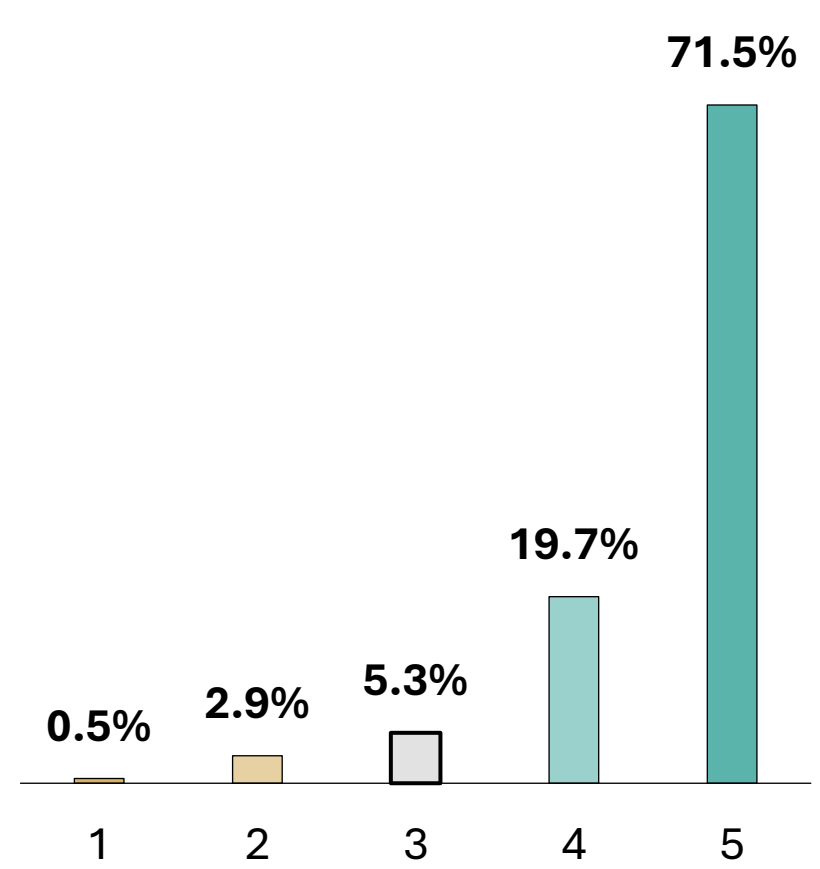 	"With some of our paramedics who are less technologically (adept), that seems to be a lot of my day-to-day, even just getting people's email passwords reset or accessing their email app or accessing their (online) schedule, all of that seems to be very challenging. So we would need a robust support system where either a supervisor could ask on behalf of a paramedic or the paramedic could receive that support very quickly and seamlessly. Because a lot of our paramedics, despite kind of how our society is moving, surprisingly, they still just think they only prioritize learning about technological advances that they want to learn about, and so when it's associated with work, they don't necessarily make that connection or put in that time or that effort." – I #10	Acce<br>for<br>criti<br>Give<br>natu<br>spe<br>reac

**Accuracy** (89%)<br>*High word recognition rates, and ability to select a best fit word from a paramedicine specific vocabulary.*	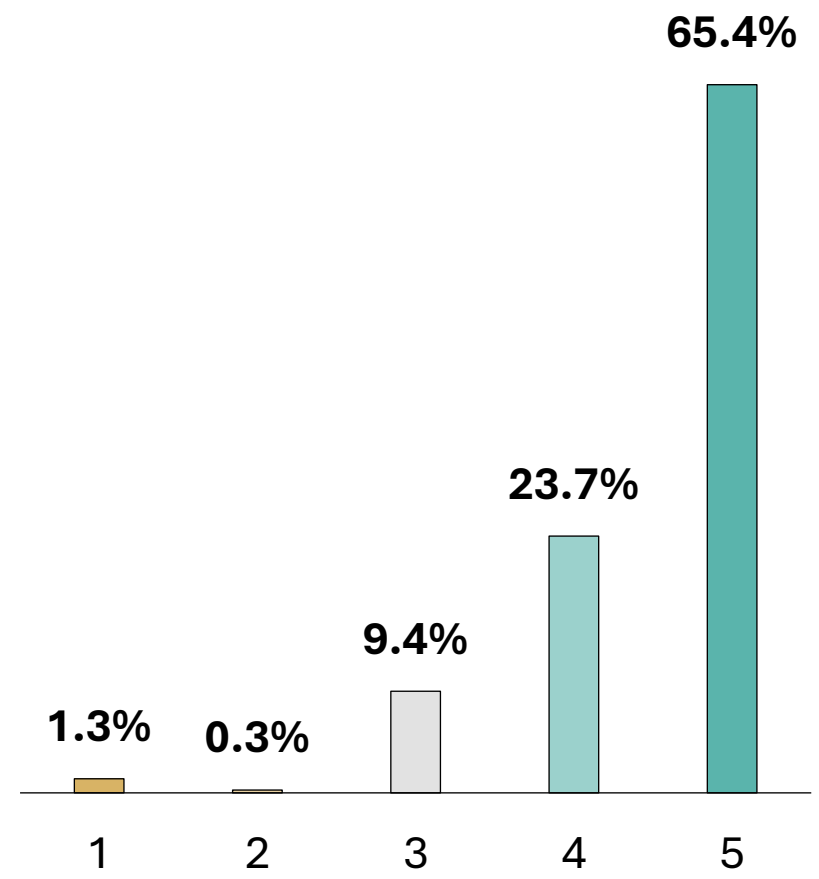	"So I think for me, the biggest thing would be, yeah, comprehension and clarity, but then also if I think that if it was transcribing my speech and it was incorrect would be very frustrating and very discouraging. I wouldn't want to use the technology. I would find it just easier to just write it out myself." – I #6<br><br>"The system will save the most time when it makes the least mistakes possible; otherwise, I'll just have to do a ton of editing afterwards." – R #161	Para<br>uniq<br>acro<br>an<br>us<br>uniq<br>hea<br><br>soft<br>accu<br>f<br>spe<br>im
**Training** (89%)<br>*Adequate training is provided, including time to practice and learn the interface.*	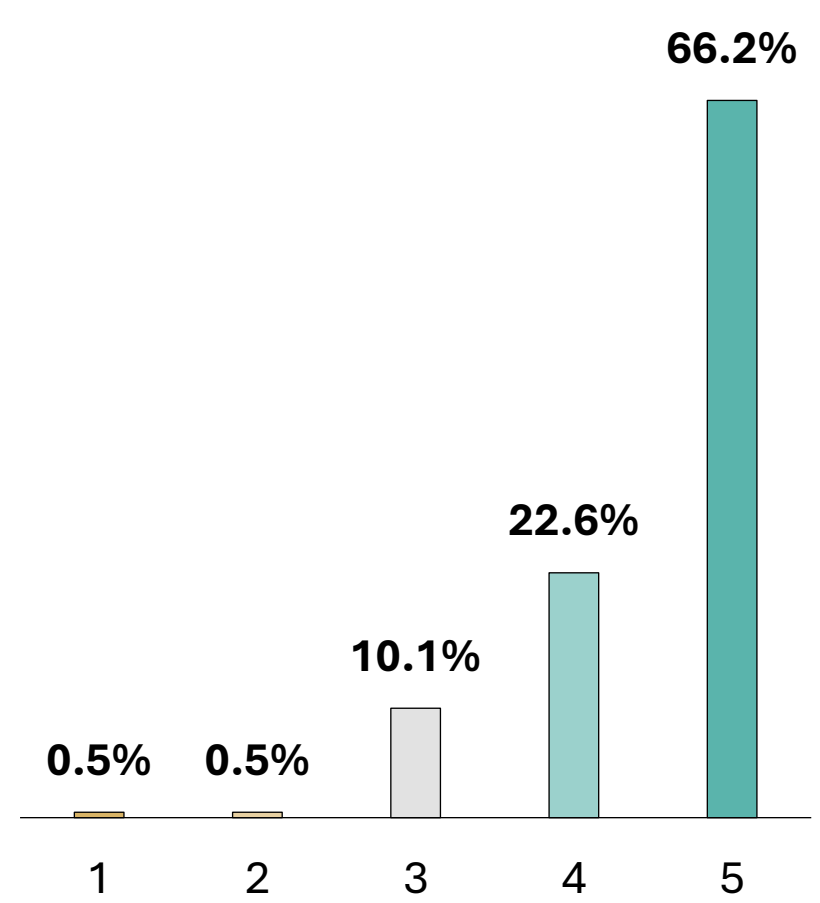	"I think definitely taking the time to have a proper training session. It seems like a lot of stuff that my service does is trying to condense it to as little training as possible. I think if this were to be successful, like a long training session and like just like an in-person training session. Really give us time to get used to it and understand how it works and the potential shortfalls and the potential benefits and stuff like that would be immensely beneficial." – I #2<br><br>"(If) We don't receive proper training, no one will be good at it, nor will I feel comfortable using it should we lack training." – R #15	Pa<br>they<br>app<br>l<br>tec<br>w<br>tra<br>rea<br>th<br>prop
**Continuous Improvement** (86%)<br>*Regular feedback session to determine areas of improvement, or identify areas for further expansion.*	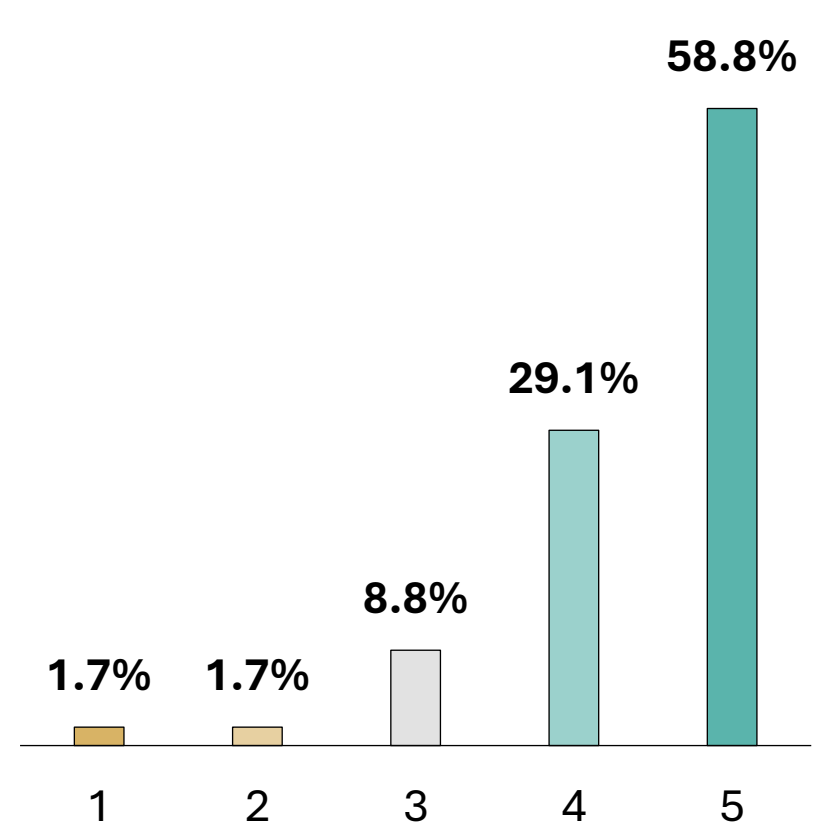	"I think definitely having a trial period and kind of figuring out if this says something that does work for us or not. We tend to do that a lot with our equipment. Just because we can figure out, okay, this is working or okay, this works, but 95% of the time, this is what's hindering our ability to use it. That kind of thing." – I #12<br><br>"That our input of what doesn't work ACTUALLY equates to change" – R #15	P<br>havi<br>Th<br>fe<br>regul<br>the<br>softv<br>for

Editing (86%)
The ability to manually edit speech recognition entries using a keyboard or digital interface.

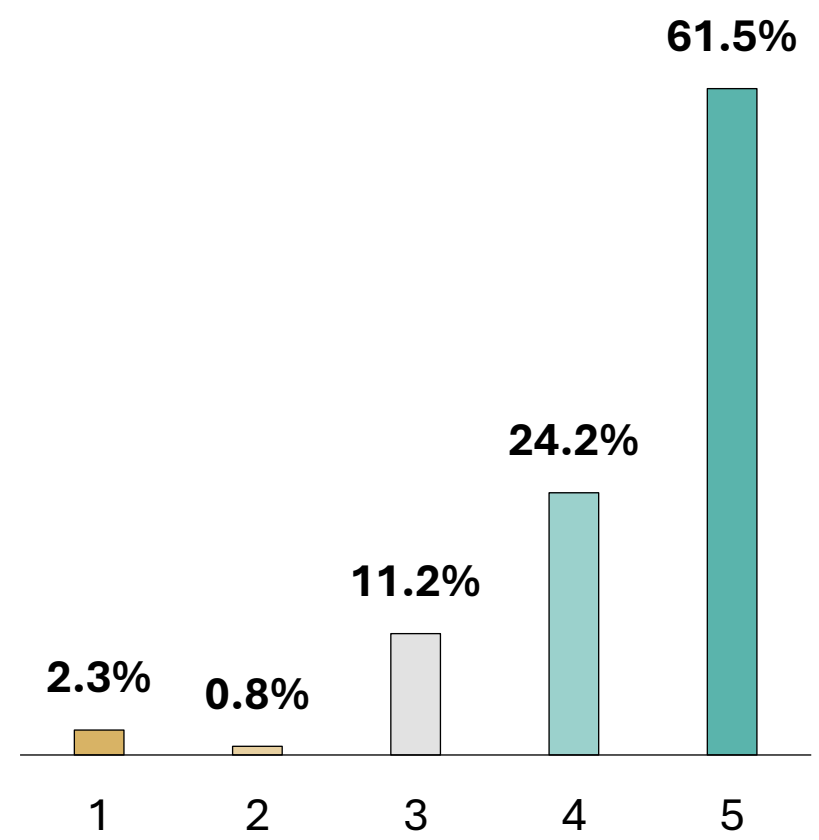

"I need the ability to edit after the call for accuracy, I want it to be smart & not have to change the way I do a call to have it recognize things" – R #288

Par
ab
fir
(

Dictation (86%)
Ability to dictate narrative documentation to the device and have that transcribed into an ePCR.

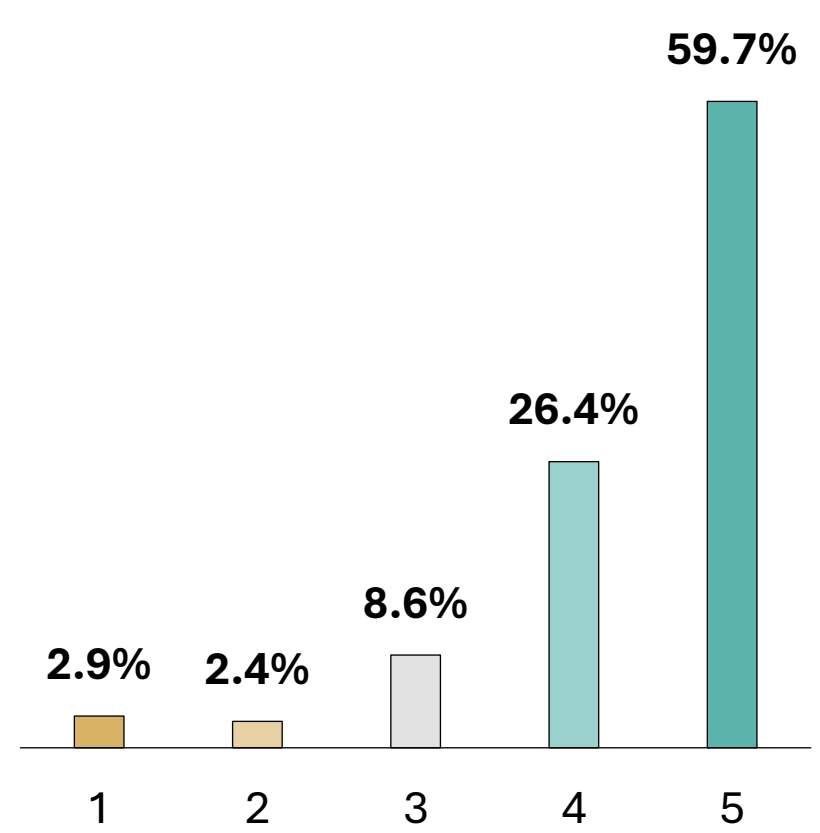

"That is a tough one cause if I look most globally, It would be speech-to-text within a chart. I can see all kinds of, you know on scene, stating things that will then upload into your chart, but I think most people struggle with getting that narrative in a period of time. So if they could speak it, I think that would have the largest impact for everyone, the whole system" – I #3

The
(
sec
w
featu
t

Training Support (86%)
Policies to support adequate training resources for the use of the speech recognition devices.

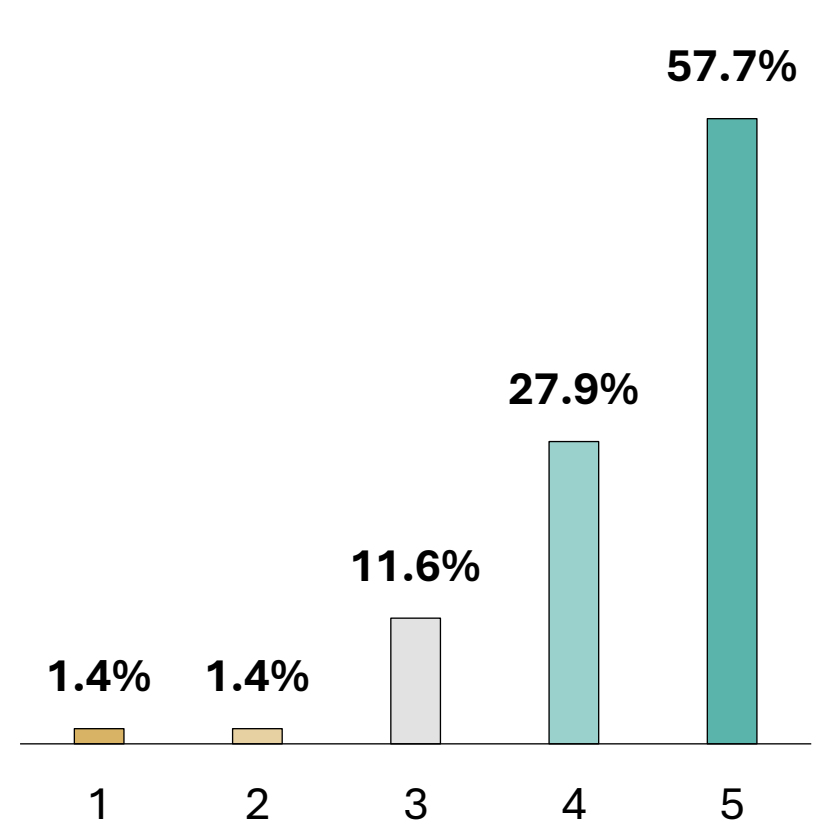

"Not to speak negatively of our organization. But I do know from experience that our organization is notoriously bad at training us on new technologies and services or whatever it is. Skills. So, there would really need to be robust training. And it's probably not something offered by the company itself. Maybe something more so by the manufacturers of the technology. The people that know it best. Kind of hear the information from the horse's mouth, so to speak. Yeah, I think that would be the number one, training." – I #19

"If time was not given (by the organization) to properly learn and integrate the technology" – R #491

Tl
pr
pres
ana
war
t
tec
the
us
war
t
with
full

APPENDIX A

Table A1. List of questions, short forms and abbreviations.

Short Title	Abbreviation	Definition
**Hardware**		
Ergonomics	Ergo	Ergonomics - The comfort of the Earpiece/Headset/Device that is worn to interface with the speech recognition software.
Durability	Dura	Durability - Sturdiness of the device, ability to easily sanitize, battery life, weatherproofing, etc.
Customizability	Cust	Customizability - Ability to change/reshape or have an individual device created.
Local Processing	LocPro	Local Processing - Ability to use speech recognition software without cellular or Wi-Fi connection.
Interoperability Devices	InterOpD	Interoperability (Devices) - Ability of software to interact with other devices (e.g., EKG monitor, IV Pump, laptop) and issue commands to devices.
Communication	Comms	Communication - Ability to use the device for dual purposes, speech recognition documentation and communication with dispatch/receiving ED.
Integration	Integrat	Integration - The ability of the device to be integrated into the ePCR system that is currently in use. I.e. not requiring a new ePCR system to use the SR device.
**Clinical Context**		
Medication History	MedHis	Medications - Ability to record patient medications through spelling name/DIN/Rx number.
Vocabulary	Vocab	Vocabulary - A robust and paramedicine-specific vocabulary and the ability to store and record context/regionally specific terms.
Dictation	Dict	Dictation - Ability to dictate narrative documentation to the device and have that transcribed into an ePCR.

Vital Sign Recording	VitSigRec	Vital sign recording - Using a voice command to import vital signs from an active monitor with a timestamp.
Intervention Timestamp	InterTimes	Interventions - Timestamping interventions using voice commands using speech recognition to record details about the intervention (e.g., record placement, size, and IV flow rate).
Medication Administration Timestamp	MedAdTime	Medication Administration - Timestamping medication administration using voice commands and speech recognition to record details about the medication administration (e.g., record time, route, dose, lot, and exp date).
Medication Scanning	MedScn	Medication scanning - Scan the medication barcode prior to administration to automatically record information or record a patient's medications automatically.
Capture Images and Video	CapImgVid	Capture Images or Videos - Capture photos or videos of patient injuries
Telemedicine	Telemed	Telemedicine - Ability to stream video directly to consultation physician or paramedic from the device.
Feedback Display Smart Glasses	FeedDSG	Feedback Display - (Smart Glasses Only) Ability to receive live feedback in heads up display (e.g., CPR rate and rhythm feedback, live vital signs, recorded interventions, etc.)
**Human-Computer Interface**		
Editing	Editing	Editing - The ability to manually edit speech recognition entries using a keyboard or digital interface.
Calibration	Calib	Calibration - The ability to calibrate the device to your specific speech patterns, thereby increasing accuracy.
Accuracy	Accuracy	Accuracy - High word recognition rates, and ability to select a best fit word from a paramedicine specific vocabulary.
Minimal Interface	MinInter	Minimal Interface - Interface, either screen (smart glasses) or headset is small and does not pose a barrier to patient interaction.
Voice Command Operation	VocComOp	Voice Commands - Voice commands to navigate ePCR, or activate accessory equipment.

Feedback	Feedback	Feedback - Ability of device to provide either audio or visual feedback for interventions (CPR rate & depth, medication timings, etc.).
Conversational Processing	ConvPro	Conversational Processing - Ability of the speech recognition technology to decipher conversational language into the ePCR. (e.g., "Okay Ms. Smith I am going to give you a medication, 2.5 mg of Morphine through the IV line we started, you may feel nausea or a warm sensation" translates to Medication -> Morphine -> Dose: 2.5mg ->Route: IV -> Timestamp).
Closed Loop	Clsdloop	Closed Loop - The device provides a repeat of what information was recorded, or an audio or visual cue that recoding was completed.
Hands Free Operation	HFOps	Hands-Free Operation - Technology can be fully operated hands free using commands or gestures (Smart glasses).
**People**		
Support	Supp	Support - Easily accessible technical support for troubleshooting issues.
Patient	Patient	Patient - The device used to interact with speech recognition must not interfere with patient interaction.
Training	Train	Training - Adequate training is provided including time to practice, learn the interface.
Operation levels	OpLvls	Operation Levels - Account for users' technological abilities. Basic - intermediate - advanced settings for a user's comfort or ability with the technology.
Stakeholders	StakeHo	Stakeholders - Involvement of receiving hospital/health authority, knowledge and systems in place to receive incoming information. Examples include, dedicated parallel repository systems in the emergency department, in hospital EHR that can receive some basic amount of paramedic data.
Customization	Custom	Customization - Ability to make adjustments to the interface that suit the users' particular needs.

**Workflow and Communication**		
Dual Users	DualUse	Dual Users - The ability for both paramedics to record information on an ePCR simultaneously.
Differentiation	Differ	Differentiation - The ability of the speech recognition technology to differentiate between active patient care and conversation between paramedic partners.
Adaptability	Adapt	Adaptability - The ability for the speech recognition technology to be integrated into existing paramedic workflows (e.g., the technology works on current ePCRs, and can be used with existing communications systems).
Interoperability Institutional	InteropIns	Interoperability (Institutional) - Ability to use speech recognition to transmit recorded patient information to the receiving facility (e.g., voice command to transmit vitals, EKGs, recorded interventions and medications).
**Organizational Policies, Procedures, and Culture**		
Training Support	TrainSup	Training Support - Policies to support adequate training resources for use of the speech recognition devices.
Stakeholder Engagement	StakHEng	Stakeholder Engagement - The organization engages hospitals, health authorities and other response agencies and creates policies to allow the integration of the technology.
Organizational Buy In	OrgBuyIn	Organizational Buy-In - Leadership within the organization has committed to the use of speech recognition technology and recognizes the benefits and time savings.
**External Rules, Regulations and Pressures**		
Compliance	Compl	Compliance - The speech recognition device meets all required medicolegal requirements for recording audio and video of patients.
Storage	Storag	Storage - The speech recognition service stores all recorded conversations in a cloud service for required period for recall when required or mandated.

Deletion	Delet	Deletion - The speech recognition software deletes all recordings from the local device as the user logs out from the system.
**System Measurement and Monitoring**		
Published Use Studies	PublishUS	Published Use Studies - Test group studies to determine the efficiency and effectiveness of the speech recognition technology in a live environment.
Continuous Improvement	ContinImp	Continuous Improvement - Regular feedback session to determine areas of improvement, or identify areas for further expansion.
User Testimonials	UserTest	User Testimonials - The ability to hear from other paramedics/paramedic services who have used similar devices or technologies.
**Technology Familiarity**		
Speech to text	StTFam	Speech to text - Function on smartphones (For text messages or search functions)
Voice Assistants	VcAssFam	Voice Assistants - (e.g., Siri, Alexa, Google Home)
Medical Dictation	MedDicFam	Medical Dictation - (e.g., Dragon ONE Nuance medical dictation, Deepscribe)
Vehicle Systems	VhclSysFam	Vehicle Systems - (e.g., OnStar, BMWs MBUX)
Voice Command Appliances	VoComAppFam	Voice Command Appliances - (e.g., XBox Kinect, Whirlpool smart oven)
Smart Glasses	SmrtGFam	Smart Glasses - (e.g., Google glass, Vuzix M400, Microsoft hololens)
**Technology Use**		
Speech to Text	StTUse	Speech to Text function on Smartphone
Voice Assistant	VcAssUse	Voice Assistant
Medical Dictation 1	MedDicUse	Medical Dictation
Vehicle System	VhclSysUse	Vehicle System
Voice Command Appliances 1	VoComAppUse	Voice Command Appliances
Smart Glasses 1	SmrtGUse	Smart Glasses
**Perceptions (What is your perception of speech recognition technology?)**		
More Accurate	MoAcc	Speech recognition documentation is more accurate than typed documentation.
More Efficient	MoEff	Speech recognition documentation takes less time to complete than typed entry.

Not Disruptive	NotDis	Speech recognition documentation would not be disruptive to my patient interactions.
More Complete Notes	MoCompN	Speech recognition documentation would help me produce more complete and accurate notes at handover that will increase patient safety.

APPENDIX B

Table B1: Phase two interview participant selection matrix.

Participant ID	Gender			Paramedicine Role						Location			Practice area			Work Experience			O. Percep.		Specific Perceptions			
	M	W	Non-B	FS	E	L/E	R	St	Sup	E	C	W	U	SU	R	0-5	6-10	11+	P	N	Td	Lt	Nd	Ma
PP01	X			X								X	X					X	X		5	4	3	4
PP02	X			X								X	X				X		X		5	5	4	5
PP03		X			X	X	X			X				X	X			X	X		3	4	2	4
PP04		X		X							X		X			X			X		1	2	1	2
PP05		X		X							X		X				X		X		2	4	3	4
PP06			X	X				X		X				X	X	X				X	2	2	1	2
PP07	X			X								X	X					X		X	2	3	1	2
PP08	X			X							X		X	X		X				X	2	4	2	3
PP09		X			X	X	X			X			X				X			X	1	3	3	3
PP10		X			X		X		X		X		X					X	X		4	5	4	5
PP12		X		X				X				X			X			X	X		3	5	1	1
PP13	X			X	X				X			X	X					X	X		4	5	4	5
PP17		X		X							X		X			X				X	3	3	2	2
PP19	X			X						X					X	X			X		4	4	4	5

Abbreviations: Gender - M (Man), W (Woman), Non-B (Non-Binary); Paramedicine Role – FS (Frontline Staff), E (Educator), L/E (Leadership/Executive), R (Researcher), St (Student), Sup (Field Supervisor); Location – E (Eastern Canada – Newfoundland, Nova Scotia, PEI, New Brunswick), C (Central Canada – Quebec, Ontario, Manitoba), W (Western Canada – Saskatchewan, Alberta, British Columbia); Practice Area – U (Urban), SU (Suburban), R (Rural); O. Precep – Overall Perception; Specific Perceptions – Td (More accurate than typed), Lt (Less time than typed), Nd (Not disruptive to patient interaction), Ma (More complete notes available).
